# Large Differences in Bacterial Community Composition among Three Nearby Extreme Waterbodies of the High Andean Plateau

**DOI:** 10.3389/fmicb.2016.00976

**Published:** 2016-06-21

**Authors:** Pablo Aguilar, Eduardo Acosta, Cristina Dorador, Ruben Sommaruga

**Affiliations:** ^1^Lake and Glacier Ecology Research Group, Institute of Ecology, University of InnsbruckInnsbruck, Austria; ^2^Laboratory of Microbial Complexity and Functional Ecology, Antofagasta Institute, University of AntofagastaAntofagasta, Chile; ^3^Centre for Biotechnology and Bioengineering, Universidad de ChileSantiago, Chile

**Keywords:** bacterial diversity, pyrosequencing, 16S rRNA gene, DOC, ponds, Salar de Huasco, Altiplano

## Abstract

The high Andean plateau or Altiplano contains different waterbodies that are subjected to extreme fluctuations in abiotic conditions on a daily and an annual scale. The bacterial diversity and community composition of those shallow waterbodies is largely unexplored, particularly, of the ponds embedded within the peatland landscape (i.e., Bofedales). Here we compare the small-scale spatial variability (<1 m) in bacterial diversity and community composition between two of those ponds with contrasting apparent color, using 454 pyrosequencing of the 16S rRNA gene. Further, we compared the results with the nearest (80 m) main lagoon in the system to elucidate the importance of different environmental factors such as salinity and the importance of these ponds as a source of shared diversity. Bacterial diversity was higher in both ponds than in the lagoon and community composition was largely different among them and characterized by very low operational taxonomic unit sharing. Whereas the “green” pond with relatively low dissolved organic carbon (DOC) concentration (33.5 mg L^-1^) was dominated by Proteobacteria and Bacteroidetes, the one with extreme DOC concentration (424.1 mg L^-1^) and red hue was dominated by Cyanobacteria. By contrast, the lagoon was largely dominated by Proteobacteria, particularly by Gammaproteobacteria. A large percentage (47%) of all reads was unclassified suggesting the existence of large undiscovered bacterial diversity. Our results suggest that even at the very small-scale spatial range considered, local environmental factors are important in explaining differences in bacterial community composition in those systems. Further, our study highlights that Altiplano peatland ponds represent a hitherto unknown source of microbial diversity.

## Introduction

Extreme ecosystems are characterized by high or low, and sometimes largely fluctuating, values of at least one environmental factor such as, for example, UV radiation, temperature, pH, salinity, or pressure (Seufferheld et al., 2008). Such environments are often dominated by microbes that show a high degree of adaptation and enable them to establish a population (Goordial et al., 2016). The Andean Altiplano is a highland plateau with an average altitude of 4000 m located between ca. 15° and 22° S (Risacher et al., 2003) and within a semi-arid region (Garreaud et al., 2003). This area presents several extreme environmental conditions including high midday solar (UV) radiation (600–1100 W m^-2^), extreme daily temperature changes (-10 to +25°C), negative water balance, low atmospheric pressure (40% lower than that at sea level), and a wide range of salinity ranges (from freshwater to saturated saltwater within the same basin; Dorador et al., 2010, 2013). There are several closed hydrographic basins in this area, which together show significant variation in physicochemical and geomorphological characteristics (Risacher et al., 2003). One of those basins, the Salar de Huasco, Chile, includes the permanent waters of the homonymous lagoon and a complex mosaic of streams, peatlands, salt crusts, and ponds (Vila et al., 2007; Dorador et al., 2013). Studies in the lagoon of Salar de Huasco have shown that this ecosystem presents a large number of novel bacterial clusters and 16S rRNA gene phylotypes, including Cyanobacteria (Dorador et al., 2008b), Archaea (Dorador et al., 2010), Bacteroidetes (Dorador et al., 2009), and ammonia-oxidizing bacteria (Dorador et al., 2008a).

Ponds are small shallow and stagnant waterbodies that hold water permanently or temporarily and have been useful to study ecological patterns of community assembly and ecoevolutionary feedbacks (De Meester et al., 2005). They can be found in environments ranging from polar deserts to tropical rainforests (Céréghino et al., 2008). Due to their patchy geographical distribution, the importance of ponds in regional- and large-scale biogeochemical studies remains elusive (Holgerson, 2015).

Microorganisms living in ponds have been identified as key components in the biogeochemical cycling of carbon, nitrogen, and other elements (Hahn, 2006). The factors that shape the bacterial community composition (BCC) of ponds and lakes have been analyzed at different spatial scales ranging from <1 to >2500 km (e.g., van der Gucht et al., 2007). This study, for example, revealed that local environmental factors are more important than spatial distance in explaining changes in BCC. Similar studies, however, at spatial scales <0.1 km are rare for stagnant freshwaters because seldom do they lie so close together. One exception are the ponds found embedded within the peatland (locally termed Bofedales) landscape in the Altiplano. Bofedales are peat-accumulating systems dominated by cushion plant communities with relatively high organic carbon production (Cooper et al., 2015).

In this study, we assessed the BCC and diversity using NGS in two nearby (<1 m) small peat ponds and compared them with that of the lagoon of Salar de Huasco, located ca. 80 m apart from the ponds. Though the ponds had a conspicuously different color, we expected to find a similar BCC considering their close proximity and the same surrounding environmental matrix (i.e., the bofefal). By contrast, we expected to find a different BCC in the lagoon due to its saline character (Dorador et al., 2008a).

## Materials and Methods

### Study Area and Sampling

**FIGURE 1:**
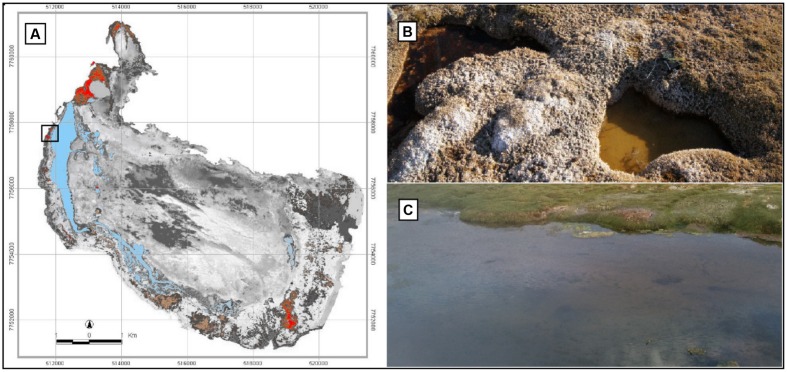
**Sampling sites at the Salar de Huasco.** **(A)** Map of Salar de Huasco showing the sampling sites at station H3 (black square). Peatlands (“Bofedales”) are showed in red/brown and water bodies in blue. **(B)** The red (left side) and green pond (right side). **(C)** The lagoon at station H3 (black square in **(A)**).

The Salar de Huasco (20°18′18 S, 68°50′22 W, Chile, **Figure 1A**) is located at 3800 m above sea level and has a mean air temperature and precipitation of 5.0°C and 150 mm year^-1^, respectively (Risacher et al., 2003). Bofedales develop where groundwater keeps the ecosystem permanently wet close to streams and lakes (Cooper et al., 2015). The water sources of these systems are freshwater and low salinity groundwater originating from glacier streams, snowmelt, and rain (Squeo et al., 2006). In August 2013 (i.e., during the dry season), water samples were collected from two shallow (ca. 20 cm depth) ponds taking care of not disturbing the sediment and avoiding algal clumps. The ponds had different apparent color (hereafter: E72-Red and E73-Green) and light penetrated to the bottom. They were embedded within the Bofedales landscape and located just <1 m apart (**Figure 1B**). Samples were also collected from the lagoon of the Salar de Huasco (hereafter: E74-Blue) close to the station H3 in Dorador et al. (2008a), which was ca. 80 m apart from the ponds (**Figure 1C**). Lamentably, due to a sudden dust storm, no more samples were possible to collect from those remote systems. Water samples were filtered *in situ* using 0.22 μm Sterivex filters (Millipore) until clogging was observed and then, the filters were stored in lysis buffer (2 ml) [50 mM Tris–HCl (pH 9), 20 mM EDTA, 400 mM NaCl, and 0.75 M sucrose], immediately placed on ice, and stored at -20°C in the laboratory until DNA extraction.

### Physicochemical Parameters

*In situ* measurements of temperature and pH were done with a portable pH meter and coupled thermometer (HI9126, Hanna Instruments), whereas electrical conductivity was measured with a portable conductivity meter (Orion Star A322, Thermo Scientific). Due to the shallowness of the systems, only one measurement was done. Samples were also collected in parallel for the analysis of major anions (nitrate, chloride, and sulfate) and cations (potassium, sodium, calcium, and magnesium) by ion chromatography (Dionex ICS-1100/1000). Additional samples were collected in precombusted (4 h at 450°C) glass bottles for the analysis of dissolved organic carbon (DOC) and dissolved nitrogen (DN). These samples were filtered *in situ* through two pre-combusted GF/F filters (Whatman). The filtrate was acidified with HCl to pH 2 and analyzed later at the laboratory in Innsbruck with a Shimadzu TOC-Vc series equipped with a total nitrogen module. An appropriate dilution (three parallels) was done with Milli-Q water of low carbon content to fit the calibration range. The instrument for DOC analysis was calibrated with potassium hydrogen phthalate, while calibration for the DN was done with potassium nitrate. Three to five subsamples were analyzed for each sample and for a consensus reference material (CRM) for DOC (batch 5 FS-2005: 0.57 mg; provided by RSMAS/MAC, University of Miami) that was run in parallel on each occasion. Results differed from the CRM given value by 5%, and the coefficient of variation among subsamples was <2%. From the same filtrate, spectral characteristics of the chromophoric dissolved organic matter (CDOM) were measured in a double-beam spectrophotometer (Hitachi). We calculated the ratio of the slopes (*S*_R_) of log-transformed absorption over two wavelength regions (275–295 and 350–400 nm) as a proxy (inversely related) of the dominant molecular weight of CDOM (Helms et al., 2008).

### DNA Extraction and 454 Pyrosequencing

Genomic DNA was extracted using a PowerBiofilm DNA Isolation kit (Mo Bio Laboratories Inc.) following the manufacturer’s protocol. The concentration and quality of DNA were measured with a Nanodrop spectrophotometer (Nanodrop 8000, Thermo Scientific). The extracted DNA (300 ng) was used as template for 16S rRNA gene fragments amplification and further pyrosequencing done at the Research and Testing Laboratory (Lubbock, TX, USA) using a Roche 454 FLX platform with the universal primers 28F (5′-GAGTTTGATCMTGGCTCAG-3′) and 519R (5′-GWATTACCG CGGCKGCTG-3′; Turner et al., 1999). Raw pyrosequencing reads have been deposited in the sequence read archive (SRA) of NCBI under accession number SRP068879.

### Data Analysis

The raw pyrosequencing dataset was processed using Mothur (v. 1.35.1) following Schloss 454 SOP protocol^1^ (Schloss et al., 2011). The Shhh.flow command was used as the first step to reduce sequencing noise, and the trim.seqs command was used to trim the reads with the average quality score of 25, and to eliminate any reads that was not in the 100–400 bp range. Reads were aligned to the SILVA-compatible alignment database using align.seqs command. A pre-cluster step was applied to further reduce sequencing noise. Chimeras were detected and removed using UCHIME. The SILVA119 database was used to classified reads with a confidence threshold of 80%. The remove.lineage commands were used to identify and remove mitochondrial, chloroplasts, Archaea, Eukarya, and unknown contaminants. Reads were assigned to operational taxonomic units (OTU) at the 3% level of divergence using the cluster.classic command. To test for differences between the water bodies, all samples were randomly subsampled to the same size according to the sample with the smallest number of reads. Rarefaction curves and alpha diversity metrics such as Chao1, Fisher, Shannon (*H*′), and Simpson (1 – *D*) including their bootstrap confidence intervals (9999) were calculated using PAST (Hammer et al., 2001). Representative reads of each OTU was compared with GenBank using BLASTn tool to determine the novelty (or uniqueness) of reads.

The Mothur shared file was converted to a Cytoscape network file using a custom R script (Neave et al., 2014). The dataset was used with the total number of OTUs, and singleton reads were removed to reduce complexity. The network was constructed as a bipartite graph, containing both OTUs and sites as nodes, and edges were drawn between OTUs and the site in which they were detected. The weight of the edge was proportional to the abundance of the OTU. The networks were visualized using Cytoscape v3.2.1^2^ Beta diversity metrics were calculated using Unifrac (Lozupone and Knight, 2005), implemented in Mothur, to assess the similarity between communities membership (unifrac.unweighted command) and structure (unifrac.weighted command). Relaxed neighbor joining phylogenetic tree was done using CLEARCUT (Evans et al., 2006) implemented in Mothur, and visualized through iTOL^3^ (Letunic and Bork, 2011).

## Results

### Physicochemical Characteristics

Water temperatures measured at midday were 15.3 and 11.3°C in E72-Red and E73-Green ponds, respectively, whereas in the lagoon (E74-Blue), it was 14.3°C. The pH in all systems was alkaline with the highest value found in pond E72-Red (8.8) followed by the lagoon E74-Blue and pond E73-Green (**Table 1**). By contrast, the conductivity was highest in the lagoon (40.35 mS cm^-1^), followed by the green (28.75 mS cm^-1^) and the red pond (8.89 mS cm^-1^). In agreement with the conductivity values, the ionic concentration followed the same trend, though there were also differences in ionic composition (**Supplementary Figure S1**). For example, the lagoon had a higher dominance of chloride than the ponds. The concentration of DOC was highest in the pond E72-Red with 424.1 mg L^-1^ and it was one order of magnitude higher than in the green pond and the lagoon (**Table 1**). DN was again highest in the pond E72-Red (19.4 mg L^-1^), but the concentration in the lagoon was higher (2.7 mg L^-1^) than in pond E73-Green (1.5 mg L^-1^). The slope ratio (*S*_R_) for CDOM (**Supplementary Figure S2**) was highest in the lagoon (2.01), followed by the ponds E73-Green (0.98) and E72-Red (0.89).

**Table 1:** Main physicochemical parameters of the three systems.

System/Code	Water temperature (°C)	pH	Conductivity 25°C (mS cm^-1^)	DOC (mg L^-1^)	DN (mg L^-1^)
Pond E72-Red	15.3	8.8	8.89	424.1	19.4
Pond E73-Green	11.3	8.1	28.75	33.5	1.5
Lagoon E74-Blue	14.3	8.7	40.35	30.4	2.7


*DN, dissolved nitrogen; DOC, dissolved organic carbon.*

### Bacterial Diversity

After quality control, we obtained 5921 reads from all samples, the highest number of reads was detected in the lagoon (3022 reads), followed by pond E73-Green (1827 reads) and pond E72-Red (1072 reads). Thus, it was subsampled to 1072 reads. The highest OTU number was recovered from pond E73-Green with a total of 371 followed by E72-Red (359) and E74-Blue samples (157). The pond E73-Green showed also the highest alpha diversity metrics in almost all indexes (**Table 2**), while the lowest were found in the lagoon. However, the extrapolated richness (Chao1) was higher in pond E72-Red. The rarefaction curves of the OTUs indicated that diversity was not completely sampled in all three systems (**Supplementary Figure S3**).

**Table 2:** Total number of reads, OTUs number, and diversity metrics for the three systems.

System/Code	Number of reads	Number of OTUs	Chao1 (CI)	Fisher’s (CI)	Shannon (CI)	Simpson (CI)
Pond E72-Red	1072	359	817 (740.2–894.3)	189.3 (177.3–201.2)	4.7 (4.6–4.9)	0.96 (0.95–0.97)
Pond E73-Green	1827	493	668 (605.5–730.5)	201 (188.8–213.1)	5.3 (5.2–5.4)	0.99 (0.98–0.99)
Lagoon E74-Blue	3022	245	311 (266.3–355.8)	50.7 (46.3–55.0)	3.5 (3.4–3.6)	0.93 (0.92–0.94)


*CI: bootstrapped confidence interval at 95%.*

### Bacterial Community Composition

**FIGURE 2:**
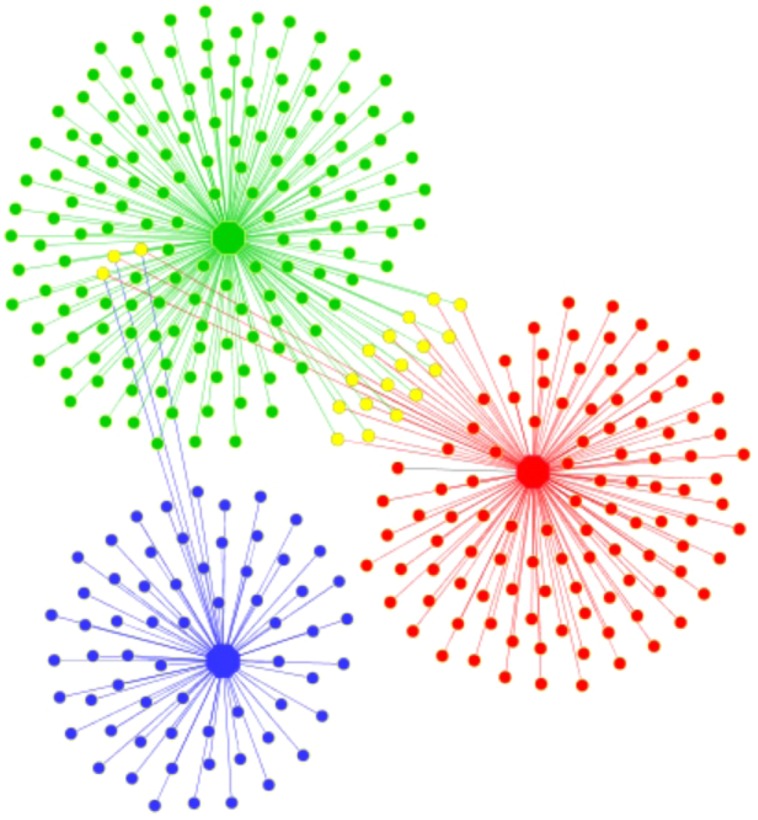
**Network analyses of operational taxonomic units (OTUs) from bacterial communities of Pond E72-Red (red dots), Pond E73-Green (green dots), and the lagoon E74-Blue (blue dots).** Shared OTUs are indicated by yellow dots.

**FIGURE 3:**
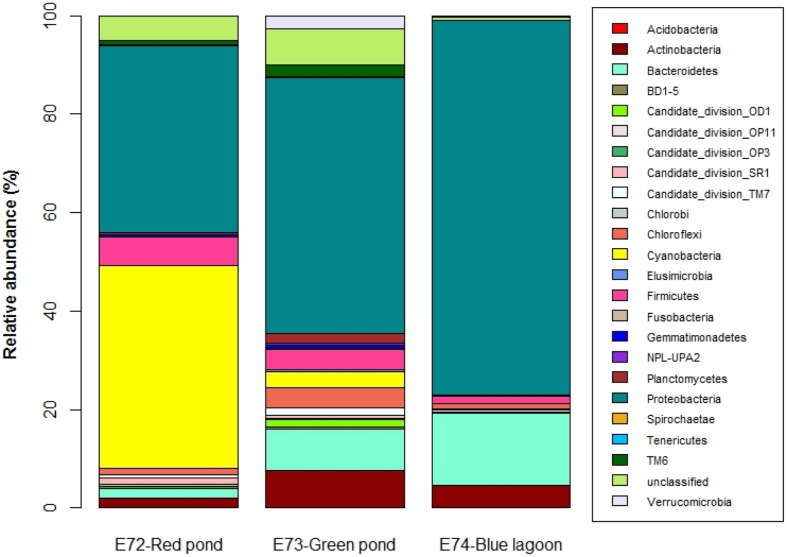
**Relative abundance of the different phyla (and candidate divisions) for the three sites**.

In general, ponds shared just a few OTUs between them, but the shared fraction between the ponds and the lagoon was even lower, showing that virtually each system had distinct bacterial communities (**Figure 2**). This was supported by the UniFrac results that showed significant differences (*P* < 0.001) in community membership and structure among samples (**Supplementary Table S1**). Only three OTUs were shared by all samples and represented by *Seohaeicola*, *Loktanella*, and *Halomonas*. The phylogenetic distribution of taxa found in the samples showed that the pond E72-Red was dominated by OTUs which were closely related (**Supplementary Figure S4**). There was no representative in this part of the phylogenetic tree that was shared with the lagoon and only a few with pond E73-Green. The latter was more evenly represented on the phylogenetic tree, which is in agreement with the higher diversity found in this system. In total, we identified 23 different bacterial phyla (**Figure 3**), though at the genus level, 47% of reads from all samples were unclassified. The most abundant phylum (% relative abundance) in pond E72-Red was Cyanobacteria (41%), followed by Proteobacteria (38.1%) and Firmicutes (5.9%). Within the reads recovered and classified as Cyanobacteria, 50% were unclassified (**Supplementary Table S2**). The filamentous cyanobacterium *Arthrospira* (15.6%) was dominant in this group of reads. Others cyanobacterial taxa identified with <2% of relative abundance were *Leptolyngbya, Oscillatoria*, and *Nodularia*. The prevailing Proteobacteria class in this pond was Alphaproteobacteria (20.4%) and 85% of this class was represented by members of Rhodobacteraceae family.

In pond E73-Green, the most abundant phylum was Proteo bacteria (52%), followed by Bacteroidetes (8.3%) and Actino bacteria (7.7%). Within Proteobacteria (**Supplementary Table S3**), the most abundant class was Alphaproteobacteria (21.4%) with *Hyphomicrobium, Rhodobacter*, and *Pedomicrobium* as domi nant taxa. The second most abundant class was Betaproteo bacteria (15%) that was mainly represented by *Hydrogenophaga* and an unclassified group. Further, we detected the presence of Gammaproteobacteria, Deltaproteobacteria, and Epsilonproteo bacteria. Within Bacteroidetes, *Flavobacterium* was the most abundant genus.

The main groups in the lagoon (E74-Blue) were Proteobacteria (76.1%), Bacteroidetes (14.5%), and Actinobacteria (4.7%). The most important taxa were the Gammaproteobacteria *Halomonas* and *Spiribacter* (**Supplementary Table S4**). All reads classified as Alphaprotebacteria (the second most abundant class) were associated to the *Rhodobacteraceae* family, though were unclassified to genus level. High percentages (94.6%) of the reads classified as Betaproteobacteria and were related to GKS98 freshwater group (Zwart et al., 2002). The main reads of Bacteroidetes were related to *Psychroflexus* and other unclassified taxa.

The comparison of all reads from our study with those in GenBank (**Supplementary Figure S5**) indicated that the pond E72-Red had the highest percentage (11.6%) of novel reads (i.e., <97% similarity), followed by the lagoon (10%), and the pond E73-Green (7.9%).

## Discussion

The three studied systems differed strongly in several physicochemical characteristics and although environmental factors have a prominent role in shaping microbial biogeographic patterns (Hanson et al., 2012), this was unexpected considering they are located next to each other, particularly, the two ponds. All systems had alkaline pH which is typical for waterbodies in the Altiplano (Marquez-Garcia et al., 2009), though the groundwater feeding the bofedales is typically acidic (Cooper et al., 2015). The different conductivity and ionic composition of the lagoon indicate that the source of water is different to that of the ponds. However, between the two close ponds, there was also a large difference (ca. threefold) in conductivity, which is probably explained by in-pond processes rather than by a different groundwater source. One particularly striking difference between the two ponds and with the lagoon was in DOC concentration. Indeed, the DOC concentration measured in pond E72-Red was far above (by 92 mg L^-1^) the highest value reported for aquatic systems (Sobek et al., 2007). Thus, this type of pond that shows disproportionately high carbon and nitrogen values compared to the surroundings can be considered biogeochemical hotspots in this area (McClain et al., 2003). What process explains the large difference in DOC concentration between two systems that are just <1 m apart is intriguing. One possibility is that it is related to the difference dominance by primary producers in these two systems. Though we did not measure chlorophyll-a as a proxy for algal biomass, visual observation of the ponds indicated that E73-Green presented suspended microalgae, while the pond E72-Red did not (but see below dominance by cyanobacteria). By contrast, in the latter pond, there was a developed benthic mat. Another possible explanation is the occurrence of animal droppings from wild vicuña (*Vicugna vicugna*) and guanaco (*Lama guanicoe*) that are inhabitants of the Altiplano (Squeo et al., 2006). Though we observed animal droppings in several areas, they were neither found inside, nor in the close surroundings of those specific ponds, at least at the time of sampling. Nevertheless, the lower slope absorbance ratios (*S*_R_) of the ponds than in the lagoon (**Supplementary Figure S2**) indicate the major influence of the peat and the predominance of high molecular weight DOM in these ponds.

Another important difference between the ponds was in their apparent water color. The red hue in pond E72 might be partially associated to high carotenoid concentrations synthesized by phototrophic organisms to minimize damage by UV radiation. This is reasonable because even if the high DOC concentration in this pond will strongly absorb these damaging wavelengths, the shallowness of the system (ca. 20 cm) and the occurrence of strong winds will expose microorganisms to the high incident solar UV radiation existing at this altitude. Another possibility is that the apparent red hue is given by the presence of iron. At the high pH observed in the pond (8.8), iron will be mostly insoluble and in colloidal form, but the high organic carbon content and the presence of a benthic mat could produce reduced conditions where iron becomes more soluble.

The different environmental characteristics of the systems were also reflected in a contrasting BCC found between the two ponds and at the same time, in comparison with that of the lagoon. Unexpectedly, the pond E72-Red was largely dominated by cyanobacteria followed by Proteobacteria (**Figure 3**). The relative dominance of cyanobacteria in this pond is atypical when compared with previous results for other water bodies in the Altiplano (Dorador et al., 2008b). A large proportion (120 out 140) of the OTUs corresponding to Cyanobacteria was unclassified suggesting a large, unexplored diversity. The known OTUs were related with *Arthrospira*, *Oscillatoria*, and *Leptolyngbya* which are also typically found in other high salinity ecosystems, such as in the Salton Sea, a lake located in the Southern California desert (Wood et al., 2002). Whether the cyanobacterial dominance in this pond (probably also in the benthic mats) could relate to the extreme DOC concentration found remains to be demonstrated. High DOC release rates by benthic cyanobacteria, however, are known (Brocke et al., 2015). Another important bacterial component in this pond was the Alphaproteobacteria, particularly members of the Rhodobacteraceae, but again with an unknown affiliation at the genus level. This family comprises aerobic photo- and chemoheterotrophs, as well as non-sulfur photosynthetic bacteria thriving in anaerobic habitats (Pujalte et al., 2014), which was not the case here.

The most abundant phyla in pond E73-Green were Bacteroidetes and Proteobacteria, the latter being the dominant phylum in the lagoon too. The same relative dominance has been observed in several water bodies of the Altiplano (Demergasso et al., 2010; Dorador et al., 2013), although direct comparison should be done with caution considering the different resolution level of the methods used (NGS vs. DGGE or clone libraries). Both *Flavobacterium* in E73-Green and *Psychroflexus* in E74-Blue were the most abundant Bacteroidetes taxa and they are reported to be common in several aquatic environments of the Altiplano (Dorador et al., 2009) and other (hypersaline lakes). For example, *Psychroflexus tropicus* is an obligately halophilic bacterium found in a Hawaiian hypersaline lake (Donachie et al., 2004). By contrast, in pond E72-Red, <2% of the reads belonged to Bacteroidetes and the few OTUs found were different from those previously known for this area (Dorador et al., 2009). It is difficult to pinpoint the cause for the different contribution of Bacteroidetes in these systems, but many members of this phylum are known to live associated with particles (Kirchman, 2002), which were present in the lagoon (high turbidity) and in the green pond (algal detritus and microalgae), but not in the red one, where only flocs at the very surface were observed. In particular, members of the *Cytophaga-Flavobacteria* are chemoorganotrophic and able to degrade complex organic polymers such as cellulose from algal origin (Kirchman, 2002).

Interestingly, in all three systems, members of the candidate division OD1 (Parcubacteria) were found suggesting the existence of suboxic conditions (Peura et al., 2012), typically found in waterbodies at this high altitude (Dorador et al., 2009) and probably even more in such high DOC environments. This phylum has recently been proposed to be symbionts of other bacteria (Nelson and Stegen, 2015). Further, it is important to highlight the presence in all samples of different OTUs considered part of the recently described candidate phyla radiation (CPR, e.g., OD1, OP11, SR1, and TM7; Brown et al., 2015) and also others described as microbial dark matter (e.g., OP3 and TM6; Rinke et al., 2013). Generally, CPR genomes are small (determined using single-cell genomics) and lack several biosynthetic pathways commonly present in bacteria, so that they seem to need other organisms for survival. Therefore, these ponds would be an interesting site to study the specific adaptations and diversity of this elusive subdivision of Bacteria.

Our results, though descriptive, suggest that even at the small spatial scale here studied, local environmental factors are crucial in shaping the BCC. The large heterogeneity observed among very close ecosystems in the Altiplano resemble that found among rock pools (Langenheder and Ragnarsson, 2007) and hydrothermal springs (Bowen De León et al., 2013), where environmental factors and historical events shape the BCC. Though, salinity is known to be a strong selective factor for bacteria (Langenheder et al., 2003), it remains to be tested what environmental factors shape the different BCC of the ponds in the bofedal. Finally, our study highlights peatland ponds of the Altiplano as a potential source of novel microbial diversity that remains to be explored. This a further reason to preserve these unique and climatically sensitive environments, which in other regions of Chile and South America are already under strong human pressure (Valdivia et al., 2013).

## Author Contributions

PA, CD, and RS, collected the samples, PA and EA prepared the samples for pyrosequencing, PA run the bioinformatic analysis, PA and RS wrote most of the manuscript, and CD and EA contributed with the writing. CD and RS obtained funding for the project. All authors have read and approved this manuscript.

## Conflict of Interest Statement

The authors declare that the research was conducted in the absence of any commercial or financial relationships that could be construed as a potential conflict of interest.

## Footnotes

1 http://www.mothur.org/wiki/454_SOP

2 http://cytoscape.org/.

3 http://itol.embl.de/
