# Supplementary material for: Large Differences in Bacterial Community Composition among Three Nearby Extreme Waterbodies of the High Andean Plateau
Source: Front Microbiol. 2016 Jun 21;7:976. doi: 10.3389/fmicb.2016.00976 (PMC4914511; doi:10.3389/fmicb.2016.00976)
Supplement: Supplementary file 1 [file Data_Sheet_1.DOCX]

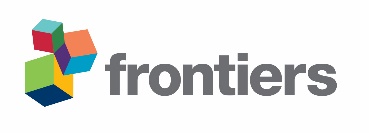


Supplementary Material

**Large differences in bacterial community composition among three nearby extreme waterbodies of the high Andean plateau**

***Pablo Aguilar^1, 2,3^, Eduardo Acosta^2,3^, Cristina Dorador^2,3^* and Ruben Sommaruga^1^****

^1^ Lake and Glacier Ecology Research Group, Institute of Ecology, University of Innsbruck, Austria.

^2^ Laboratory of Microbial Complexity and Functional Ecology, Antofagasta Institute, University of Antofagasta, Chile.

^3^Centre for Biotechnology and Bioengineering (CeBiB).

*** Correspondence**:

Ruben Sommaruga, Lake and Glacier Ecology Research Group, Institute of Ecology, University of Innsbruck, Technikerstr. 25, 6020 Innsbruck, Austria. ruben.sommaruga@uibk.ac.at.

and

Cristina Dorador, Departamento de Biotecnología, Facultad de Ciencias del Mar y Recursos Biológicos, Instituto Antofagasta, Universidad de Antofagasta, Av. Angamos 601, Antofagasta, Chile. cristina.dorador@uantof.cl

## Supplementary Tables

**Supplementary Table S1**. Results of the UniFrac analysis using unweighted and weighted options, as well as corresponding p values.

**Supplementary Table S2.** Summary of the most abundant (79.1%) bacterial taxa in the pond E72-Red.

|  |  |  |  |  |  |  |  |  |
| --- | --- | --- | --- | --- | --- | --- | --- | --- |
|  |  |  | **Rank** |  |  | **Name** | **%** |  |
|  | Phylum |  |  |  |  | **Cyanobacteria** | **41.0** |  |
|  |  | Class |  |  |  | **Cyanobacteria** | **20.5** |  |
|  |  |  | Order |  |  | **Subsection I** | **0.1** |  |
|  |  |  |  | Family |  | **Family I** | **0.1** |  |
|  |  |  |  |  | Genus | **Unclassified** | **0.1** |  |
|  |  |  | Order |  |  | **Subsection III** | **19.0** |  |
|  |  |  |  | Family |  | **Family I** | **19.0** |  |
|  |  |  |  |  | Genus | ***Arthrospira*** | **15.6** |  |
|  |  |  |  |  | Genus | ***Leptolyngbya*** | **0.5** |  |
|  |  |  |  |  | Genus | ***Oscillatoria*** | **2.0** |  |
|  |  |  |  |  | Genus | ***Spirulina*** | **0.7** |  |
|  |  |  |  |  | Genus | **Unclassified** | **0.2** |  |
|  |  |  | Order |  |  | **Subsection IV** | **1.2** |  |
|  |  |  |  | Family |  | **Family I** | **1.2** |  |
|  |  |  |  |  | Genus | ***Nodularia*** | **0.3** |  |
|  |  |  |  |  | Genus | **Unclassified** | **0.9** |  |
|  |  |  | Order |  |  | **Unclassified** | **0.2** |  |
|  |  | Class |  |  |  | **Unclassified** | **20.5** |  |
|  | Phylum |  |  |  |  | **Proteobacteria** | **38.1** |  |
|  |  | Class |  |  |  | **Alphaproteobacteria** | **20.4** |  |
|  |  |  | Order |  |  | **DB1-14** | **0.5** |  |
|  |  |  |  | Family |  | **Unclassified** | **0.5** |  |
|  |  |  | Order |  |  | **Rhizobiales** | **1.3** |  |
|  |  |  |  | Family |  | **Hyphomicrobiaceae** | **1.2** |  |
|  |  |  |  |  | Genus | ***Hyphomicrobium*** | **0.7** |  |
|  |  |  |  |  | Genus | ***Pedomicrobium*** | **0.5** |  |
|  |  |  |  | Family |  | **Rhodobiaceae** | **0.1** |  |
|  |  |  |  |  | Genus | ***Rhodobium*** | **0.1** |  |
|  |  |  | Order |  |  | **Rhodobacterales** | **17.4** |  |
|  |  |  |  | Family |  | **Rhodobacteraceae** | **17.4** |  |
|  |  |  |  |  | Genus | ***Gemmobacter*** | **7.7** |  |
|  |  |  |  |  | Genus | ***Loktanella*** | **3.5** |  |
|  |  |  |  |  | Genus | ***Rhodobaca*** | **0.5** |  |
|  |  |  |  |  | Genus | ***Roseibaca*** | **0.2** |  |
|  |  |  |  |  | Genus | ***Seohaeicola*** | **2.0** |  |
|  |  |  |  |  | Genus | **Unclassified** | **3.5** |  |
|  |  |  | Order |  |  | **Rhodospirillales** | **0.2** |  |
|  |  |  |  | Family |  | **Unclassified** | **0.2** |  |
|  |  |  | Order |  |  | **Rickettsiales** | **0.3** |  |
|  |  |  |  | Family |  | **LWSR-14** | **0.1** |  |
|  |  |  |  |  | Genus | **Unclassified** | **0.1** |  |
|  |  |  |  | Family |  | **Rickettsiales_incertae_sedis** | **0.2** |  |
|  |  |  |  |  | Genus | **Candidatus_captivus** | **0.2** |  |
|  |  |  | Order |  |  | **Sphingomonadales** | **0.1** |  |
|  |  |  |  | Family |  | **Sphingomonadaceae** | **0.1** |  |
|  |  |  |  |  | Genus | ***Sandarakinorhabdus*** | **0.1** |  |
|  |  |  | Order |  |  | **Unclassified** | **0.7** |  |
|  |  | Class |  |  |  | **Betaproteobacteria** | **3.5** |  |
|  |  |  | Order |  |  | **Burkholderiales** | **3.4** |  |
|  |  |  |  | Family |  | **Comamonadaceae** | **3.4** |  |
|  |  |  |  |  | Genus | ***Hydrogenophaga*** | **2.6** |  |
|  |  |  |  |  | Genus | **Unclassified** | **0.8** |  |
|  |  |  | Order |  |  | **Nitrosomonadales** | **0.1** |  |
|  |  |  |  | Family |  | **Nitrosomonadaceae** | **0.1** |  |
|  |  |  |  |  | Genus | **Unclassified** | **0.1** |  |
|  |  |  | Order |  |  | **Unclassified** | **0.1** |  |
|  |  | Class |  |  |  | **Epsilonproteobacteria** | **5.0** |  |
|  |  |  | Order |  |  | **Campylobacterales** | **5.0** |  |
|  |  |  |  | Family |  | **Campylobacteraceae** | **0.3** |  |
|  |  |  |  |  | Genus | ***Arcobacter*** | **0.3** |  |
|  |  |  |  | Family |  | **Helicobacteraceae** | **4.7** |  |
|  |  |  |  |  | Genus | ***Sulfurimonas*** | **4.7** |  |
|  |  | Class |  |  |  | **Gammaproteobacteria** | **8.7** |  |
|  |  |  | Order |  |  | **Aeromonadales** | **0.1** |  |
|  |  |  |  | Family |  | **Aeromonadaceae** | **0.1** |  |
|  |  |  |  |  | Genus | ***Aeromonas*** | **0.1** |  |
|  |  |  | Order |  |  | **Chromatiales** | **1.1** |  |
|  |  |  |  | Family |  | **Chromatiaceae** | **0.9** |  |
|  |  |  |  |  | Genus | ***Allochromatium*** | **0.1** |  |
|  |  |  |  |  | Genus | ***Thioalkalicoccus*** | **0.1** |  |
|  |  |  |  |  | Genus | ***Thiocapsa*** | **0.5** |  |
|  |  |  |  |  | Genus | **Unclassified** | **0.2** |  |
|  |  |  |  | Family |  | **Ectothiorhodospiraceae** | **0.2** |  |
|  |  |  |  |  | Genus | ***Ectothiorhodospira*** | **0.2** |  |
|  |  |  | Order |  |  | **Legionellales** | **2.3** |  |
|  |  |  |  | Family |  | **Coxiellaceae** | **1.6** |  |
|  |  |  |  |  | Genus | ***Aquicella*** | **0.8** |  |
|  |  |  |  |  | Genus | ***Coxiella*** | **0.8** |  |
|  |  |  |  | Family |  | **Legionellaceae** | **0.7** |  |
|  |  |  |  |  | Genus | ***Legionella*** | **0.7** |  |
|  |  |  | Order |  |  | **Oceanospirillales** | **2.1** |  |
|  |  |  |  | Family |  | **Halomonadaceae** | **0.3** |  |
|  |  |  |  |  | Genus | ***Halomonas*** | **0.3** |  |
|  |  |  |  | Family |  | **Oceanospirillaceae** | **1.9** |  |
|  |  |  |  |  | Genus | ***Nitrincola*** | **1.8** |  |
|  |  |  |  |  | Genus | ***Pseudospirillum*** | **0.1** |  |
|  |  |  | Order |  |  | **Thiotrichales** | **1.8** |  |
|  |  |  |  | Family |  | **Piscirickettsiaceae** | **1.7** |  |
|  |  |  |  |  | Genus | **Unclassified** | **1.7** |  |
|  |  |  |  | Family |  | **Unclassified** | **0.1** |  |
|  |  |  | Order |  |  | **Unclassified** | **0.9** |  |
|  |  |  | Order |  |  | **Xanthomonadales** | **0.3** |  |
|  |  |  |  | Family |  | **Xanthomonadaceae** | **0.3** |  |
|  |  |  |  |  | Genus | ***Aquimonas*** | **0.3** |  |
|  |  | Class |  |  |  | **Unclassified** | **0.5** |  |
|  |  |  |  |  |  |  |  |  |

**Supplementary Table S3.** Summary of the most abundant (60.3%) bacterial taxa in the pond E73-Green.

|  |  |  |  |  |  |  |  |
| --- | --- | --- | --- | --- | --- | --- | --- |
|  |  |  | **Rank** |  |  | **Name** | **%** |
|  | Phylum |  |  |  |  | **Proteobacteria** | **52.0** |
|  |  | Class |  |  |  | **Alphaproteobacteria** | **21.4** |
|  |  |  | Order |  |  | **Caulobacterales** | **0.2** |
|  |  |  |  | Family |  | **Caulobacteraceae** | **0.2** |
|  |  |  |  |  | Genus | ***Brevundimonas*** | **0.2** |
|  |  |  | Order |  |  | **DB1-14** | **1.2** |
|  |  |  |  | Family |  | **Unclassified** | **1.2** |
|  |  |  | Order |  |  | **Rhizobiales** | **8.8** |
|  |  |  |  | Family |  | **C2U** | **0.3** |
|  |  |  |  |  | Genus | **Unclassified** | **0.3** |
|  |  |  |  | Family |  | **Hyphomicrobiaceae** | **8.0** |
|  |  |  |  |  | Genus | ***Devosia*** | **0.7** |
|  |  |  |  |  | Genus | ***Hyphomicrobium*** | **4.5** |
|  |  |  |  |  | Genus | ***Pedomicrobium*** | **2.4** |
|  |  |  |  |  | Genus | ***Rhodomicrobium*** | **0.1** |
|  |  |  |  |  | Genus | **Unclassified** | **0.3** |
|  |  |  |  | Family |  | **MNG7** | **0.2** |
|  |  |  |  |  | Genus | **Unclassified** | **0.2** |
|  |  |  |  | Family |  | **Rhizobiales_Incertae_Sertis** | **0.1** |
|  |  |  |  |  | Genus | ***Bauldia*** | **0.1** |
|  |  |  |  | Family |  | **Xanthobacteraceae** | **0.2** |
|  |  |  |  |  | Genus | ***Pseudolabrys*** | **0.2** |
|  |  |  | Order |  |  | **Rhodobacterales** | **7.6** |
|  |  |  |  | Family |  | **Rhodobacteraceae** | **7.6** |
|  |  |  |  |  | Genus | ***Loktanella*** | **0.3** |
|  |  |  |  |  | Genus | ***Oceanicella*** | **0.2** |
|  |  |  |  |  | Genus | ***Paracoccus*** | **0.3** |
|  |  |  |  |  | Genus | ***Rhodobacter*** | **1.7** |
|  |  |  |  |  | Genus | ***Tropicimonas*** | **0.3** |
|  |  |  |  |  | Genus | **Unclassified** | **4.9** |
|  |  |  | Order |  |  | **Rhodospirillales** | **1.6** |
|  |  |  |  | Family |  | **Rhodospirillaceae** | **0.7** |
|  |  |  |  |  | Genus | ***Rhodospirillum*** | **0.7** |
|  |  |  |  | Family |  | **Rhodospirillales_Incertae_Sedis** | **0.7** |
|  |  |  |  |  | Genus | ***Reyranella*** | **0.7** |
|  |  |  |  | Family |  | **Unclassified** | **0.1** |
|  |  |  | Order |  |  | **Rickettsiales** | **0.7** |
|  |  |  |  | Family |  | **Holosporaceae** | **0.2** |
|  |  |  |  |  | Genus | **Unclassified** | **0.2** |
|  |  |  |  | Family |  | **Rickettsiales_Incertae_Sedis** | **0.5** |
|  |  |  |  |  | Genus | **Candidatus_Odyssella** | **0.5** |
|  |  |  |  | Family |  | **Unclassified** | **0.1** |
|  |  |  | Order |  |  | **Sphingomonadales** | **0.2** |
|  |  |  |  | Family |  | **Erythrobacteraceae** | **0.2** |
|  |  |  |  |  | Genus | ***Porphyrobacter*** | **0.2** |
|  |  |  | Order |  |  | **Unclassified** | **1.2** |
|  |  | Class |  |  |  | **Betaproteobacteria** | **15.0** |
|  |  |  | Order |  |  | **Burkholderiales** | **14.3** |
|  |  |  |  | Family |  | **Alcaligenaceae** | **0.6** |
|  |  |  |  |  | Genus | **GKS98_freshwater_group** | **0.1** |
|  |  |  |  |  | Genus | **unclassified** | **0.5** |
|  |  |  |  | Family |  | **Comamonadaceae** | **13.8** |
|  |  |  |  |  | Genus | ***Hydrogenophaga*** | **7.0** |
|  |  |  |  |  | Genus | ***Paucibacter*** | **0.1** |
|  |  |  |  |  | Genus | ***Pelomonas*** | **0.3** |
|  |  |  |  |  | Genus | ***Rhodoferax*** | **0.1** |
|  |  |  |  |  | Genus | **Unclassified** | **6.3** |
|  |  |  | Order |  |  | **Methylophilales** | **0.2** |
|  |  |  |  | Family |  | **Methylophilaceae** | **0.2** |
|  |  |  |  |  | Genus | **Unclassified** | **0.2** |
|  |  |  | Order |  |  | **Unclassified** | **0.3** |
|  |  | Class |  |  |  | **Deltaproteobacteria** | **2.1** |
|  |  |  | Order |  |  | **Bdellovibrionales** | **0.9** |
|  |  |  |  | Family |  | **Bacteriovoracaceae** | **0.1** |
|  |  |  |  |  | Genus | **Peredibacter** | **0.1** |
|  |  |  |  | Family |  | **Bdellovibrionaceae** | **0.8** |
|  |  |  |  |  | Genus | ***Bdellovibrio*** | **0.8** |
|  |  |  | Order |  |  | **Desulfobacterales** | **0.1** |
|  |  |  |  | Family |  | **Desulfobacteraceae** | **0.1** |
|  |  |  |  |  | Genus | **Unclassified** | **0.1** |
|  |  |  | Order |  |  | **Desulfovibrionales** | **0.2** |
|  |  |  |  | Family |  | **Desulfonatronaceae** | **0.2** |
|  |  |  |  |  | Genus | ***Desulfonatronum*** | **0.2** |
|  |  |  | Order |  |  | **Myxococcales** | **0.9** |
|  |  |  |  | Family |  | **0319-6G20** | **0.8** |
|  |  |  |  |  | Genus | **Unclassified** | **0.8** |
|  |  |  |  | Family |  | **Phaselicystidaceae** | **0.1** |
|  |  |  |  |  | Genus | ***Phaselicystis*** | **0.1** |
|  |  | Class |  |  |  | **Epsilonproteobacteria** | **0.6** |
|  |  |  | Order |  |  | **Campylobacterales** | **0.6** |
|  |  |  |  | Family |  | **Campylobacteraceae** | **0.6** |
|  |  |  |  |  | Genus | ***Arcobacter*** | **0.6** |
|  |  | Class |  |  |  | **Gammaproteobacteria** | **11.8** |
|  |  |  | Order |  |  | **Aromonadales** | **1.0** |
|  |  |  |  | Family |  | **Aeromonadaceae** | **1.0** |
|  |  |  |  |  | Genus | ***Aeromonas*** | **1.0** |
|  |  |  | Order |  |  | **Alteromonadales** | **0.1** |
|  |  |  |  | Family |  | **Alteromonadaceae** | **0.1** |
|  |  |  |  |  | Genus | **Unclassified** | **0.1** |
|  |  |  | Order |  |  | **Chromatiales** | **1.3** |
|  |  |  |  | Family |  | **Chromatiaceae** | **1.2** |
|  |  |  |  |  | Genus | ***Rheinheimera*** | **0.3** |
|  |  |  |  |  | Genus | ***Thiocapsa*** | **0.8** |
|  |  |  |  |  | Genus | **Unclassified** | **0.1** |
|  |  |  |  | Family |  | **Ectothiorhodospiraceae** | **0.1** |
|  |  |  |  |  | Genus | ***Ectothiorhodospira*** | **0.1** |
|  |  |  | Order |  |  | **Enterobacteriales** | **0.3** |
|  |  |  |  | Family |  | **Enterobacteriaceae** | **0.3** |
|  |  |  |  |  | Genus | ***Yersinia*** | **0.3** |
|  |  |  | Order |  |  | **Legionellales** | **5.0** |
|  |  |  |  | Family |  | **Coxiellaceae** | **1.5** |
|  |  |  |  |  | Genus | ***Aquicella*** | **0.75** |
|  |  |  |  |  | Genus | ***Coxiella*** | **0.75** |
|  |  |  |  | Family |  | **Legionellaceae** | **3.4** |
|  |  |  |  |  | Genus | ***Legionella*** | **3.4** |
|  |  |  | Order |  |  | **NKB5** | **1.0** |
|  |  |  |  | Family |  | **Unclassified** | **1.0** |
|  |  |  | Order |  |  | **Oceanospirillales** | **0.3** |
|  |  |  |  | Family |  | **Halomonadaceae** | **0.3** |
|  |  |  |  |  | Genus | ***Halomonas*** | **0.3** |
|  |  |  | Order |  |  | **Pseudomonadales** | **0.4** |
|  |  |  |  | Family |  | **Pseudomonadaceae** | **0.4** |
|  |  |  |  |  | Genus | ***Pseudomonas*** | **0.3** |
|  |  |  |  |  | Genus | **Unclassified** | **0.1** |
|  |  |  | Order |  |  | **Thiotrichales** | **0.7** |
|  |  |  |  | Family |  | **Piscirickettsiaceae** | **0.2** |
|  |  |  |  |  | Genus | **Methylophaga** | **0.2** |
|  |  |  |  | Family |  | **Thiotrichaceae** | **0.6** |
|  |  |  |  |  | Genus | ***Thiothrix*** | **0.6** |
|  |  |  | Order |  |  | **Unclassified** | **1.6** |
|  |  |  | Order |  |  | **Xanthomonadales** | **0.1** |
|  |  |  |  | Family |  | **Xanthomonadales_Incertae_Sedis** | **0.1** |
|  |  |  |  |  | Genus | ***Steroidobacter*** | **0.1** |
|  |  | Class |  |  |  | **TA18** | **0.5** |
|  |  |  | Order |  |  | **Unclassified** | **0.5** |
|  |  | Class |  |  |  | **Unclassified** | **0.8** |
|  | Phylum |  |  |  |  | **Bacteroidetes** | **8.3** |
|  |  | Class |  |  |  | **Bacteroidia** | **0.5** |
|  |  |  | Order |  |  | **Bacteroidales** | **0.5** |
|  |  |  |  | Family |  | **ML635J-40_aquatic_group** | **0.3** |
|  |  |  |  |  | Genus | **Unclassified** | **0.3** |
|  |  |  |  | Family |  | **Porphyromonadaceae** | **0.1** |
|  |  |  |  |  | Genus | ***Paludibacter*** | **0.1** |
|  |  |  |  | Family |  | **Rikenellaceae** | **0.1** |
|  |  |  |  |  | Genus | **vadinBC27_wastewater-sludge_group** | **0.1** |
|  |  | Class |  |  |  | **Cytophagia** | **1.4** |
|  |  |  | Order |  |  | **Cytophagales** | **1.4** |
|  |  |  |  | Family |  | **Cyclobacteriaceae** | **0.7** |
|  |  |  |  |  | Genus | ***Algoriphagus*** | **0.2** |
|  |  |  |  |  | Genus | **Unclassified** | **0.5** |
|  |  |  |  | Family |  | **Cytophagaceae** | **0.8** |
|  |  |  |  |  | Genus | ***Dyadobacter*** | **0.5** |
|  |  |  |  |  | Genus | **unclassified** | **0.3** |
|  |  | Class |  |  |  | **Flavobacteriia** | **4.7** |
|  |  |  | Order |  |  | **Flavobacteriales** | **4.7** |
|  |  |  |  | Family |  | **Flavobacteriaceae** | **4.7** |
|  |  |  |  |  | Genus | ***Arenibacter*** | **0.5** |
|  |  |  |  |  | Genus | ***Flavobacterium*** | **2.9** |
|  |  |  |  |  | Genus | ***Subsaxibacter*** | **0.8** |
|  |  |  |  |  | Genus | **unclassified** | **0.6** |
|  |  | Class |  |  |  | **Sphingobacteriia** | **1.2** |
|  |  |  | Order |  |  | **Sphingobacteriales** | **1.2** |
|  |  |  |  | Family |  | **Chitinophagaceae** | **0.4** |
|  |  |  |  |  | Genus | ***Ferruginibacter*** | **0.4** |
|  |  |  |  | Family |  | **env.OPS_17** | **0.1** |
|  |  |  |  |  | Genus | **Unclassified** | **0.1** |
|  |  |  |  | Family |  | **NS11-12_marine_group** | **0.6** |
|  |  |  |  |  | Genus | **Unclassified** | **0.6** |
|  |  |  |  | Family |  | **Unclassified** | **0.1** |
|  |  | Class |  |  |  | **Unclassified** | **0.4** |
|  |  | Class |  |  |  | **VadinHA17** | **0.3** |
|  |  |  | Order |  |  | **Unclassified** | **0.3** |
|  |  |  |  |  |  |  |  |

**Supplementary Table S4.** Summary of the most abundant (90.6%) bacterial taxa in the lagoon E74-Blue.

|  |  |  |  |  |  |  |  |  |
| --- | --- | --- | --- | --- | --- | --- | --- | --- |
|  |  |  | **Rank** |  |  | **Name** | **%** |  |
|  | Phylum |  |  |  |  | **Proteobacteria** | **76.1** |  |
|  |  | Class |  |  |  | **Alphaproteobacteria** | **26.8** |  |
|  |  |  | Order |  |  | **Rhizobiales** | **0.4** |  |
|  |  |  |  | Family |  | **Bradyrhizobiaceae** | **0.1** |  |
|  |  |  |  |  | Genus | ***Nitrobacter*** | **0.1** |  |
|  |  |  |  | Family |  | **DUNssu044** | **0.3** |  |
|  |  |  |  |  | Genus | **Unclassified** | **0.3** |  |
|  |  |  | Order |  |  | **Rhodobacterales** | **26.1** |  |
|  |  |  |  | Family |  | **Rhodobacteraceae** | **26.1** |  |
|  |  |  |  |  | Genus | ***Loktanella*** | **4.0** |  |
|  |  |  |  |  | Genus | ***Seohaeicola*** | **0.3** |  |
|  |  |  |  |  | Genus | ***Sulfitobacter*** | **0.5** |  |
|  |  |  |  |  | Genus | **Unclassified** | **21.1** |  |
|  |  |  | Order |  |  | **Rhodospirillales** | **0.2** |  |
|  |  |  |  | Family |  | **Rhodospirillales_Incertae_Sedis** | **0.2** |  |
|  |  |  |  |  | Genus | **Candidatus_Alysiosphaera** | **0.2** |  |
|  |  |  | Order |  |  | **Unclassified** | **0.1** |  |
|  |  | Class |  |  |  | **Betaproteobacteria** | **15.6** |  |
|  |  |  | Order |  |  | **Burkholderiales** | **15.6** |  |
|  |  |  |  | Family |  | **Alcaligenaceae** | **14.9** |  |
|  |  |  |  |  | Genus | **GKS98_freshwater_group** | **14.7** |  |
|  |  |  |  |  | Genus | **Unclassified** | **0.2** |  |
|  |  |  |  | Family |  | **Comamonadaceae** | **0.7** |  |
|  |  |  |  |  | Genus | ***Acidovorax*** | **0.6** |  |
|  |  |  |  |  | Genus | ***Hydrogenophaga*** | **0.1** |  |
|  |  | Class |  |  |  | **Deltaproteobacteria** | **1.5** |  |
|  |  |  | Order |  |  | **Bdellovibrionales** | **0.2** |  |
|  |  |  |  | Family |  | **Bacteriovoraceae** | **0.2** |  |
|  |  |  |  |  | Genus | ***Peredibacter*** | **0.2** |  |
|  |  |  | Order |  |  | **Desulfobacterales** | **1.2** |  |
|  |  |  |  | Family |  | **Desulfobacteraceae** | **0.6** |  |
|  |  |  |  |  | Genus | ***Desulfosalsimonas*** | **0.1** |  |
|  |  |  |  |  | Genus | ***Desulfotignum*** | **0.4** |  |
|  |  |  |  |  | Genus | **Unclassified** | **0.1** |  |
|  |  |  |  | Family |  | **Desulfobulbaceae** | **0.6** |  |
|  |  |  |  |  | Genus | ***Desulfolobus*** | **0.1** |  |
|  |  |  |  |  | Genus | ***Desulfocapsa*** | **0.1** |  |
|  |  |  |  |  | Genus | ***Desulfopila*** | **0.4** |  |
|  |  |  | Order |  |  | **Desulfovibrionales** | **0.1** |  |
|  |  |  |  | Family |  | **Desulfomicrobiaceae** | **0.1** |  |
|  |  |  |  |  | Genus | ***Desulfomicrobium*** | **0.1** |  |
|  |  | Class |  |  |  | **Epsilonproteobacteria** | **0.8** |  |
|  |  |  | Order |  |  | **Campylobacterales** | **0.8** |  |
|  |  |  |  | Family |  | **Campylobacteraceae** | **0.8** |  |
|  |  |  |  |  | Genus | ***Arcobacter*** | **0.7** |  |
|  |  |  |  |  | Genus | ***Sulfurospirillum*** | **0.1** |  |
|  |  | Class |  |  |  | **Gammaproteobacteria** | **31.3** |  |
|  |  |  | Order |  |  | **Alteromonadales** | **0.7** |  |
|  |  |  |  | Family |  | **Alteromonadaceae** | **0.7** |  |
|  |  |  |  |  | Genus | ***Haliea*** | **0.4** |  |
|  |  |  |  |  | Genus | ***Marinobacter*** | **0.1** |  |
|  |  |  |  |  | Genus | **Unclassified** | **0.2** |  |
|  |  |  | Order |  |  | **Chromatiales** | **6.8** |  |
|  |  |  |  | Family |  | **Chromatiaceae** | **0.1** |  |
|  |  |  |  |  | Genus | ***Thiocapsa*** | **0.1** |  |
|  |  |  |  | Family |  | **Ectothiohodospiraceae** | **6.7** |  |
|  |  |  |  |  | Genus | ***Spiribacter*** | **5.7** |  |
|  |  |  |  |  | Genus | ***Thioalkalivibrio*** | **0.8** |  |
|  |  |  |  |  | Genus | **Unclassified** | **0.2** |  |
|  |  |  | Order |  |  | **Oceanospirillales** | **18** |  |
|  |  |  |  | Family |  | **Halomonadaceae** | **17.8** |  |
|  |  |  |  |  | Genus | ***Halomonas*** | **17.8** |  |
|  |  |  |  | Family |  | **Oceanospirillaceae** | **0.1** |  |
|  |  |  |  |  | Genus | ***Nitrincola*** | **0.1** |  |
|  |  |  |  | Family |  | **OM182_clade** | **0.1** |  |
|  |  |  |  |  | Genus | **Unclassified** | **0.1** |  |
|  |  |  | Order |  |  | **Pseudomonadales** | **0.4** |  |
|  |  |  |  | Family |  | **Moraxellaceae** | **0.3** |  |
|  |  |  |  |  | Genus | ***Psychrobacter*** | **0.3** |  |
|  |  |  |  | Family |  | **Pseudomonadaceae** | **0.1** |  |
|  |  |  |  |  | Genus | ***Pseudomonas*** | **0.1** |  |
|  |  |  | Order |  |  | **Thiotrichales** | **0.4** |  |
|  |  |  |  | Family |  | **Francisellaceae** | **0.4** |  |
|  |  |  |  |  | Genus | ***Francisella*** | **0.4** |  |
|  |  |  | Order |  |  | **Unclassified** | **5.1** |  |
|  |  | Class |  |  |  | **Unclassified** | **0.1** |  |
|  | Phylum |  |  |  |  | **Bacteroidetes** | **13.3** |  |
|  |  | Class |  |  |  | **Bacteroidia** | **0.1** |  |
|  |  |  | Order |  |  | **Bacteroidales** | **0.1** |  |
|  |  |  |  | Family |  | **ML635J-40_aquatic_group** | **0.1** |  |
|  |  |  |  |  | Genus | **Unclassified** | **0.1** |  |
|  |  | Class |  |  |  | **Cytophagia** | **2.4** |  |
|  |  |  | Order |  |  | **Cytophagales** | **0.2** |  |
|  |  |  |  | Family |  | **Cytophagaceae** | **0.2** |  |
|  |  |  |  |  | Genus | ***Flexibacter*** | **0.2** |  |
|  |  |  | Order |  |  | **Order III** | **2.2** |  |
|  |  |  |  | Family |  | **ML301M-34** | **0.2** |  |
|  |  |  |  |  | Genus | **Unclassified** | **0.2** |  |
|  |  |  |  | Family |  | **Unclassified** | **2.0** |  |
|  |  | Class |  |  |  | **Flavobacteriia** | **11.0** |  |
|  |  |  | Order |  |  | **Flavobacteriales** | **11.0** |  |
|  |  |  |  | Family |  | **Cryomophaceae** | **0.7** |  |
|  |  |  |  |  | Genus | ***Owenweeksia*** | **0.7** |  |
|  |  |  |  | Family |  | **Flavobacteriaceae** | **10.3** |  |
|  |  |  |  |  | Genus | ***Nonlabens*** | **0.1** |  |
|  |  |  |  |  | Genus | ***Psychroflexus*** | **9.0** |  |
|  |  |  |  |  | Genus | **Unclassified** | **1.2** |  |
|  |  |  |  | Family |  | **Unclassified** | **1.0** |  |
|  |  | Class |  |  |  | **Unclassified** | **1.0** |  |
|  |  |  |  |  |  |  |  |  |

**SUPPLEMENTARY FIGURES**

**
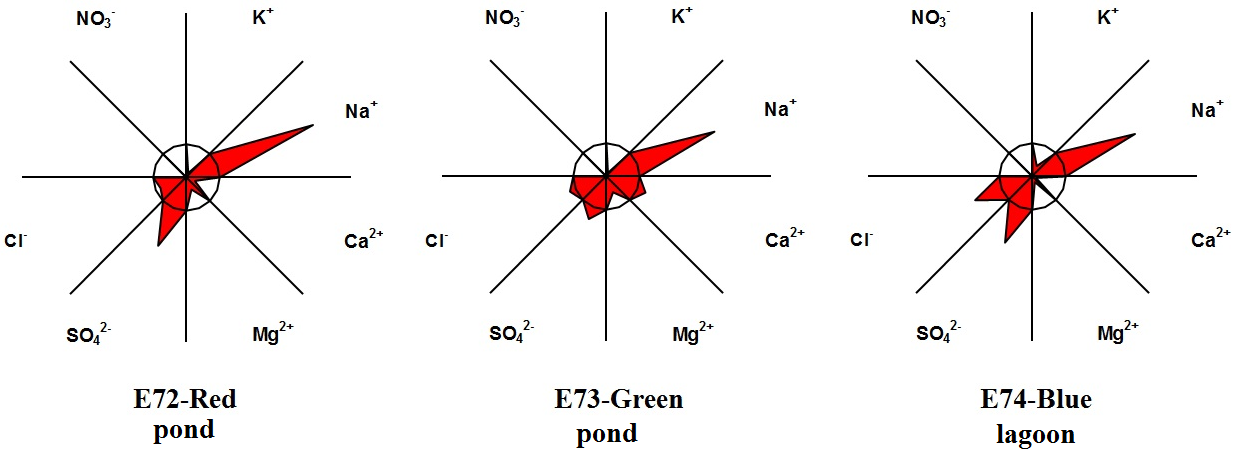
**

**Supplementary Fig. S1** Maucha diagrams showing the ionic composition of the three systems.

**Supplementary Fig. S2.** Spectral CDOM absorbance characteristics of the three systems.

**
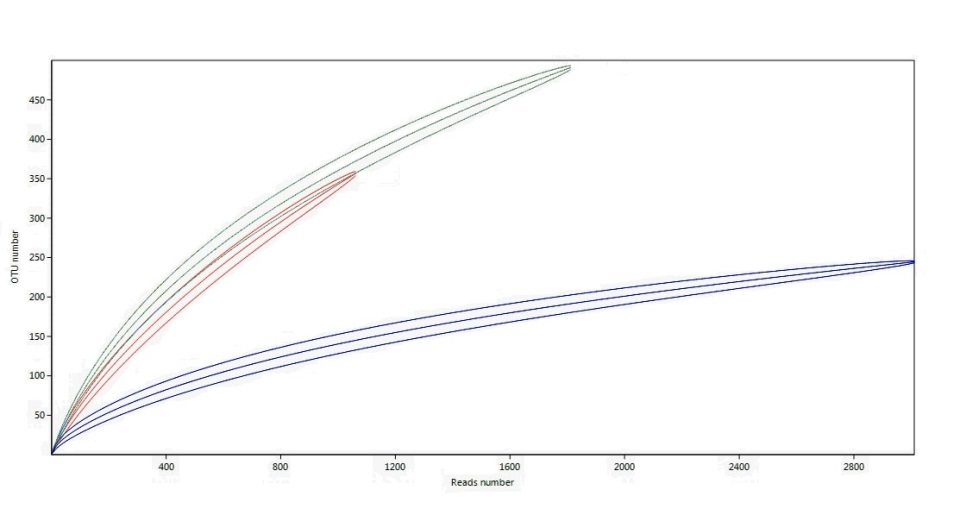
**

**Supplementary Fig. S3.** Rarefaction curves obtained from pyrosequencing data of Pond E72-Red (red lines), Pond E73-Green (green lines) and the lagoon E74-Blue (blue lines), including bootstrap confidence intervals.

.


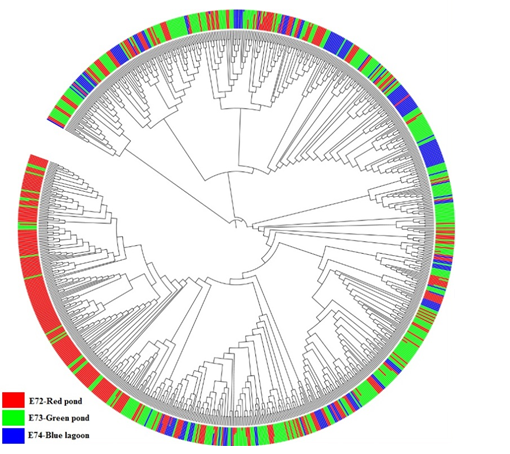


**Supplementary Fig. S4.** Phylogenetic relationship of taxa found in the three systems. The barcharts in the outer ring show the relative abundance of OTUs. Note that some OTUs occur in two samples (i.e., the bar charts have two colors). The tree was prepared using function clearcut in mothur and implemented in iTOL (branch lengths are ignored).


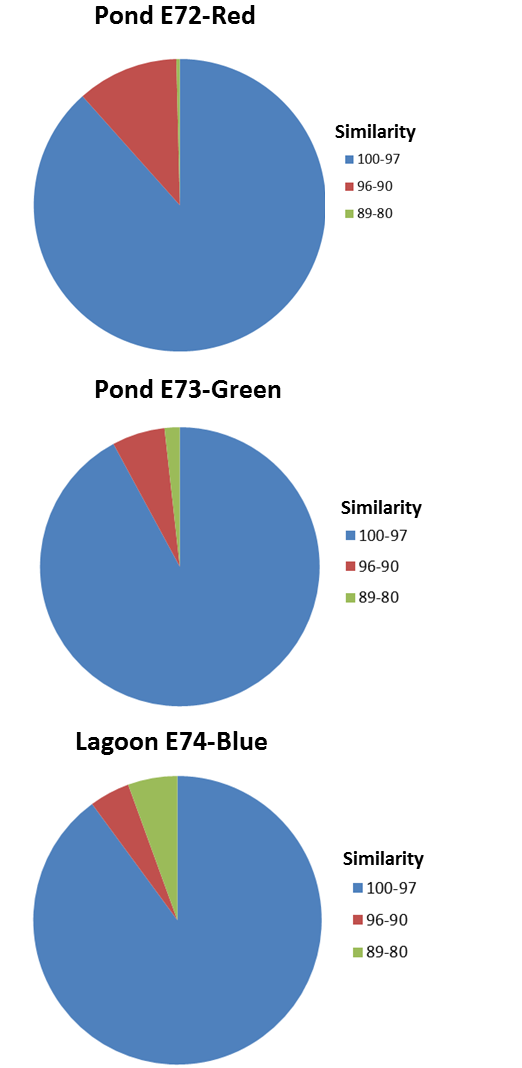


**Supplementary Fig. S5.** Percentage of reads in three different similarity ranges after comparison with Genbank using BLASTn tool to determine their novelty (or uniqueness) in the three systems. Sequences with < 97% similarity are considered novel.
